# Enhanced Discriminative Fear Learning of Phobia-Irrelevant Stimuli in Spider-Fearful Individuals

**DOI:** 10.3389/fnbeh.2014.00328

**Published:** 2014-10-01

**Authors:** Carina Mosig, Christian J. Merz, Cornelia Mohr, Dirk Adolph, Oliver T. Wolf, Silvia Schneider, Jürgen Margraf, Armin Zlomuzica

**Affiliations:** ^1^Mental Health Research and Treatment Center, Ruhr-University Bochum, Bochum, Germany; ^2^Department of Cognitive Psychology, Institute of Cognitive Neuroscience, Ruhr-University Bochum, Bochum, Germany

**Keywords:** differential fear conditioning, anxiety disorders, specific phobia, spider fear, conditionability, extinction, fear renewal, virtual reality

## Abstract

Avoidance is considered as a central hallmark of all anxiety disorders. The acquisition and expression of avoidance, which leads to the maintenance and exacerbation of pathological fear is closely linked to Pavlovian and operant conditioning processes. Changes in conditionability might represent a key feature of all anxiety disorders but the exact nature of these alterations might vary across different disorders. To date, no information is available on specific changes in conditionability for disorder-irrelevant stimuli in specific phobia (SP). The first aim of this study was to investigate changes in fear acquisition and extinction in spider-fearful individuals as compared to non-fearful participants by using the *de novo* fear conditioning paradigm. Secondly, we aimed to determine whether differences in the magnitude of context-dependent fear retrieval exist between spider-fearful and non-fearful individuals. Our findings point to an enhanced fear discrimination in spider-fearful individuals as compared to non-fearful individuals at both the physiological and subjective level. The enhanced fear discrimination in spider-fearful individuals was neither mediated by increased state anxiety, depression, nor stress tension. Spider-fearful individuals displayed no changes in extinction learning and/or fear retrieval. Surprisingly, we found no evidence for context-dependent modulation of fear retrieval in either group. Here, we provide first evidence that spider-fearful individuals show an enhanced discriminative fear learning of phobia-irrelevant (*de novo*) stimuli. Our findings provide novel insights into the role of fear acquisition and expression for the development and maintenance of maladaptive responses in the course of SP.

## Introduction

Patients with anxiety disorders and stressor-related disorders exhibit an increased avoidance of fear-related stimuli and situations. An increased tendency to avoid novel situations might constitute an important risk factor for the development and maintenance of clinical anxiety as shown in anxiety vulnerable individuals (e.g., behaviorally inhibited individuals, Fox et al., 2005) and animal models of anxiety vulnerability (Beck et al., 2010). Findings from these studies emphasized the importance of increased conditionability as a functional mechanism contributing to a strong avoidance behavior (Ricart et al., 2011; Myers et al., 2012; Holloway et al., 2014). Conditionability refers to the capacity to acquire new associations between a neutral (conditioned) stimulus (CS) and an aversive (unconditioned) stimulus (UCS) or outcome. Conditionability also comprises the ability to extinguish this association if it becomes invalid (CS-noUCS). Evidence from psychophysiological, behavioral, and imaging studies showed that individuals with high trait anxiety (Caulfield et al., 2013), patients with anxiety disorders (Lissek et al., 2005) as well as traumatized individuals (Milad et al., 2009; Norrholm et al., 2011; Jovanovic et al., 2013; Stevens et al., 2013) show systematic changes in the acquisition and extinction of conditioned fear.

Although a great deal of different methods has been utilized [see Lissek et al. (2005)], these studies typically assessed conditionability in a differential fear conditioning paradigm. Here, conditioned responses (CR) are operationalized as the difference of responses to aversively paired CS^+^ and unpaired CS^−^ as measured on the psychophysiological [e.g., skin conductance responses (SCRs), startle amplitudes] and/or subjective level (shock expectancy and subjective valence ratings) (Hermans et al., 2002; Arnaudova et al., 2013).

Given that the fear-inducing stimuli and situations as well as the associated symptoms vary between different anxiety disorders, the *de novo* fear conditioning paradigm (where participants are conditioned to unfamiliar and disorder-irrelevant stimuli) has been employed to detect alterations in general conditionability in patients with anxiety disorders and stressor-related disorders. A stronger acquisition (Orr et al., 2000; Norrholm et al., 2011) as well as a delayed extinction (Peri et al., 2000; Blechert et al., 2007) was found in patients diagnosed with post-traumatic stress disorder (PTSD) as compared to participants without trauma exposure and to healthy controls, respectively. The delayed extinction as indicated on the psychophysiological level and the level of UCS-expectancy ratings is paralleled by a weaker extinction of conditioned negative valence in PTSD (Blechert et al., 2007). In contrast, patients with panic disorder (PD) showed no differences in CR during acquisition compared to control participants (Grillon et al., 1994; Michael et al., 2007), but displayed larger SCRs to CS^+^ stimuli during extinction (Michael et al., 2007).

Overall, these findings imply that fear learning as measured on the behavioral, psychophysiological, and neuronal level is specifically altered in anxiety and stressor-related disorders and might represent a key feature of these disorders. However, there is also evidence for clear differences in fear conditioning between PD (Grillon et al., 1994; Michael et al., 2007) and PTSD patients (Orr et al., 2000; Blechert et al., 2007; Milad et al., 2009; Norrholm et al., 2011; Jovanovic et al., 2013). This suggests that changes in the ability to acquire and extinguish conditioned fear might be disorder-specific and might resemble some core symptomatic features characteristic of a certain disorder. To allow for a more general conclusion, however, comparison to yet another disorder group would be valuable. Given that the pathogenesis of SP is likely to involve cued fear conditioning, individuals with a SP would be an appropriate comparison group. SP is characterized by exaggerated fear of specific objects or situations, and cued conditioning is thought to play a central role in the etiology of this condition (e.g., Grillon, 2002). Presently, only little information is available about possible changes in general fear conditionability for *de novo* stimuli in spider-fearful individuals (Schweckendiek et al., 2011). The investigation of a group that shows a specific fear of spiders might provide valuable information on the integrity of the fear conditioning system in individuals with SP that would allow predictions on the speed of fear extinction through exposure therapy. It would also allow for comparison on differences in the magnitude and characteristics of fear learning between different forms of anxiety. For instance, the symptomatology of individuals showing a cue-specific fear (e.g., spider fear) is quite different relative to the symptomatology of individuals suffering from PTSD or PD. Besides such differences in symptomatology, there are also substantial differences between PTSD and PD on the one hand and SP on the other hand with respect to psychophysiological (Cuthbert et al., 2003; Lang and McTeague, 2009) and neuronal reactivity (Rauch et al., 2003; Etkin and Wager, 2007) during the processing of neutral and negative stimuli. Furthermore, in contrast to PD and PTSD patients, SP is associated with lower levels of anxiety and depressive symptoms (Cook et al., 1988; Cuthbert et al., 2003). Acute stress exposure (Merz et al., 2013), higher levels of tension-stress (Arnaudova et al., 2013), as well as increased anxiety levels (Dibbets et al., 2014) are linked to deficits in discriminatory fear learning. This poses another potential problem with the interpretation of previous findings on fear conditioning in clinical anxiety samples (e.g., Blechert et al., 2007; Michael et al., 2007) because differences in conditionability might be confounded by comorbid depressive symptoms and/or differences in stress levels.

In recent years, the examination of contextual effects on fear conditioning processes has become a matter of extensive clinical research because findings from these studies bear the potential to optimize exposure-based therapies in anxiety disorders (Craske et al., 2014). With respect to the treatment of anxiety disorders, the extinction of a learned association or CR leading to maladaptive behavior is equally important as learning new behavior-outcome associations, which support appropriate or “normal” behavior. Therefore, exposure-based therapy seems to be primarily based on fear extinction learning (Michael et al., 2009; Vervliet et al., 2013; Craske et al., 2014). However, extinction is a complex multi-level process. Conditioned fear responses can reoccur after extinction learning over time (spontaneous fear recovery) or when an excitatory CS is presented in an unfamiliar context (fear renewal) (Bouton, 2004, 2006). Renewal after extinction learning in experimental settings corresponds to one form of relapse after exposure therapy (Rachman, 1989; Craske et al., 2014), representing a serious problem in psychotherapy (Laborda et al., 2011). Despite the high clinical relevance, significant demonstrations of fear renewal after successful exposure therapy have been scarce so far and have yielded conflicting results (e.g., Mineka et al., 1999; Mystkowski et al., 2002, 2006). Our present knowledge of the underlying behavioral and neurobiological mechanisms governing context-dependent conditioning is primarily based on findings from animal studies and/or studies with healthy human participants (Bouton and Bolles, 1979; Bouton, 1988, 1991, 1994; Bouton and Nelson, 1998; Milad et al., 2005). To the best of our knowledge, however, there is a lack of studies assessing context-dependent fear conditioning in patients with anxiety disorders or in individuals with high levels of trait anxiety.

A promising tool for the study of contextual influences on fear conditioning is virtual reality (VR) technology (Grillon et al., 2006; Alvarez et al., 2007; Huff et al., 2011; Dunsmoor et al., 2014). The VR approach allows for systematic manipulation of context conditions and is more likely to induce a strong fear renewal since participants are provided with multisensory input in an experimental setup that more closely corresponds to real-world experiences (Huff et al., 2011). Thus, besides high ecological validity, VR techniques offer the possibility to conduct translational research on contextual effects during fear conditioning. For instance, VR environments to a great extent resemble physical multisensory contexts implemented in animal studies, as participants, in a manner analogous to rodent exploratory behavior, are engaged in the exploration of the VR environment (Huff et al., 2011). This is especially important with regard to the cross-species translational approaches examining context-dependent fear conditioning in animals and humans (Soliman et al., 2010; Haaker et al., 2013).

The present study sought to examine whether spider-fearful individuals would show alterations in the acquisition and extinction of conditioned fear. These findings could help to disentangle whether possible alterations in fear conditioning processes in participants with a specific fear of spiders are different relative to findings obtained in PTSD (Blechert et al., 2007) and PD (Michael et al., 2007). To allow for some comparability across studies, we examined differential fear conditioning in spider-fearful participants by using a modified version of the recently used differential fear conditioning paradigm (Blechert et al., 2007, 2008; Michael et al., 2007). Our paradigm utilizes the simultaneous assessment of CR on the autonomic (SCRs) and cognitive (UCS-expectancy ratings), but also the affective (valence ratings) level (Hermans et al., 2002; Blechert et al., 2007, 2008; Michael et al., 2007).

Clear differences in the amount of comorbid depression and anxiety symptoms exist across different anxiety and stressor-related disorders (Cook et al., 1988; Cuthbert et al., 2003), which might influence discriminative fear learning processes (Otto et al., 2007; Gazendam and Kindt, 2012; Arnaudova et al., 2013). Therefore, we used the Depression Anxiety Stress Scales (DASS) to control for possible effects of negative emotional states, such as anxiety, stress, and depression, on fear conditioning in spider-fearful individuals. The DASS has recently been shown to provide valuable information on the link between negative emotional states and inter-individual variability in discriminative fear learning [see Arnaudova et al. (2013)].

Given that extinction is a highly context-dependent process, another aim of this study was to determine whether spider-fearful individuals show differences in the context-dependent re-emergence of fear responses as compared to non-fearful individuals. We used VR environments as external contexts, as has previously been shown (e.g., Alvarez et al., 2007; Huff et al., 2011; Dunsmoor et al., 2014), and assessed context-dependent retrieval of extinguished CR at the subjective (expectancy and valence ratings of CS) and psychophysiological level (SCRs).

## Materials and Methods

### Participants

Individuals with a specific fear of spiders and non-fearful individuals were recruited to participate in a study dealing with “clinical implications of spider fear”. Recruiting was performed via bulletin board notices on the campus of the Ruhr-University Bochum (Germany) and by postings in social media networks. All participants were further screened using the Fear of Spiders Questionnaire [FSQ; Szymanski and O’Donohue, 1995; German version by Rinck et al. (2002)]. Only participants who explicitly reported a moderate to severe specific fear of spiders on the FSQ [cut-off score >15, according to Cochrane et al. (2008)] were assigned to the spider-fearful group. Individuals who explicitly reported to have no fear of spiders and in the FSQ scored below the cut-off were assigned to the non-fearful group. Exclusion criteria for both groups included a severe acute or chronic disease, current pharmacological or behavioral treatment for mental disease, drug/alcohol abuse or dependence, or other use of medications.

Three participants were excluded from data analyses due to technical errors during the experimental procedure. Our final sample consisted of 43 participants: 25 spider-fearful participants (mean age of 24.1, SD = 5.8) and 18 non-fearful individuals (mean age of 23.4, SD = 2.7), with a mean FSQ score of 61.1 (SD = 21.1) and 2.8 (SD = 3.2), respectively (see Table 1). All participants provided written informed consent. The study was approved by the local ethics committee of the Ruhr-University Bochum and conducted according to the guidelines of the Declaration of Helsinki. Each participant received a payment of 20€ as reimbursement.

**Table 1:** **Demographic and psychometric characteristics of spider-fearful and non-fearful participants**.

	Spider-fearful group, *M* (SD)	Non-fearful group, *M* (SD)
Age (years)	24.1 (5.8)	23.4 (2.7)
DASS (depression)	2.9 (2.7)	1.7 (2.3)
DASS (anxiety)	3.2 (2.8)	1.6 (2.7)
DASS (stress)	7.3 (4.6)	5.4 (4.6)
DASS total	13.5 (8.6)	8.7 (8.1)
FSQ total	61.1 (21.1)	2.8 (3.2)**
SPQ total	18.1 (5.1)	4.0 (2.6)**
UCS intensity (mA)	4.7 (4.1)	10.6 (6.5)*
UCS rating (−2 to +2)	−1.8 (0.5)	−1.7 (0.5)

*DASS, depression anxiety stress scale; FSQ, fear of spiders questionnaire; SPQ, spider phobia questionnaire; UCS, unconditioned stimulus*.

*Groups differed from each other in *post hoc* tests (*p* < 0.01); **Groups differed from each other in *post hoc* tests (*p* < 0.001); *T* test for independent groups.

### Experimental design

We used an adapted version of the differential fear conditioning paradigm previously developed by Blechert et al. (2007). In particular, differential fear conditioning was assessed by using a set of different dependent measures including SCRs, as well as affective (valence ratings) and cognitive (UCS-expectancy ratings) responses [see Blechert et al. (2007)]. A high-frequency tone (300 Hz) and a low-frequency tone (135 Hz) served as CS^+^ and CS^−^. CSs were counterbalanced and presented via headphones (60 dB). The presentation of CS^+^ lasted for 8 s and co-terminated with the UCS. The UCS was a mild electrical stimulation applied to the skin of the lower arm for the duration of 500 ms. The CS^−^ was never paired with the UCS. The conditioning task consisted of a habituation, acquisition, extinction, and a retrieval phase (both in the former acquisition and extinction context). During all phases, the sequence of CSs was pseudorandom, although owed to the constraint that only two identical CSs may occur consecutively. The inter-stimulus interval (ISI) was set randomly at 18–22 s.

VR software was used to examine the effects of contextual change during the phases of fear acquisition and extinction. After habituation, each participant was subjected to the entire conditioning procedure within a VR-based format. We used an AB (AB) renewal setup with a within-subject design [according to Alvarez et al. (2007)]. Each participant experienced fear acquisition in context A, but extinction was conducted in context B. Subsequently, participants were re-exposed to contexts A and B for a retrieval test. The order of presentation of context A and context B was matched across the participants. Context presentation during the acquisition and extinction phase was counterbalanced across participants and groups (for half of the participants context A served as the acquisition context and context B as the extinction context and vice versa for the other half). Also, the order of context presentation during the fear retrieval test was counterbalanced (i.e., half of the participants was returned to context A first and then entered context B, while for the other half the context order was reversed).

**Figure 1:**
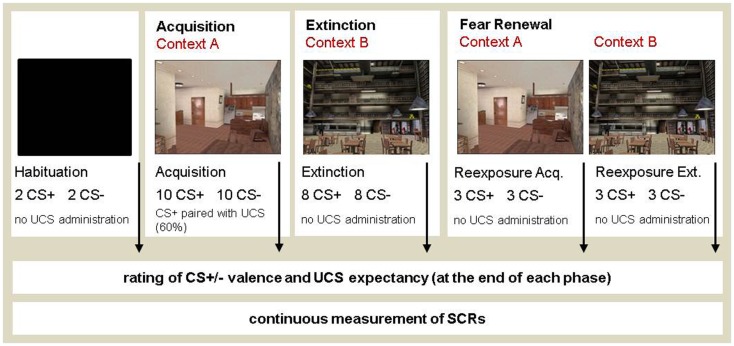
**The experimental design of the context-dependent differential fear conditioning procedure**. VR software was used for the operationalization of external context change during the phases of fear acquisition and extinction. Context 1 featured an apartment and context 2 showed a cafeteria. Participants were instructed to freely explore the VR contexts, which rotated in simultaneous correspondence to the participants’ head movements, so that they became fully immersed in the virtual context. The order of context presentation was matched across participants and counterbalanced during fear retrieval in contexts A (ret A) and B (ret B).

Max Payne software was used to create VR contexts (see Cyberpsychology Lab, University of Quebec, Outaouais, http://w3.uqo.ca/cyberpsy/en/index_en.htm). The VR environment was presented with a 3D head-mounted display (Z800, eMagin, USA). During the conditioning procedure, two different contexts were presented while the CSs were delivered via headphones (see Figure 1). The fear conditioning experiment consisted of three sessions with a break of 15 min in-between sessions. The first session consisted of a habituation phase and the acquisition phase and lasted about 15 min. Habituation served the purpose of reducing orienting responses to the CSs and to allow participants to acclimate to the experimental environment. During habituation, two CS^+^ and two CS^−^ were presented while the head-mounted display depicted only a black screen. During acquisition, a total of 10 CS^+^ and 10 CS^−^ were presented in context A. Six out of the 10 CS^+^ were paired with the UCS. In the second session, participants were extinguished in context B. Both CSs were presented eight times each, but were never paired with the UCS. The second session lasted about 10 min. In the third session, the fear retrieval test was run in both context A and context B. In each context, 3 CS^+^ and 3 CS^−^ were presented. The UCS was not administered during the fear retrieval phase.

### Apparatus and physiological recordings

The experiment was conducted in a sound-attenuated room electrically connected to an adjacent control room where the experimental apparatus was stationed. Experimenter and participant were able to communicate via headphones and microphone. A constant current electrical stimulator delivered the UCS via Ag/AgCl electrodes placed on the left lower arm of the participant. SCRs were measured via 5-mm inner diameter Ag/AgCl electrodes that were filled with non-hydrating electrode paste and attached on the distal phalanxes of the index and middle finger of the non-dominant hand. Stimulus delivery was controlled with Presentation software (Neurobehavioral Systems, USA). Physiological data was obtained in a continuous mode using a 16-bit Brain Amp ExG amplifier and was analyzed with Brain Vision Recorder Software (Brain Products, Gilching, Germany).

### Assessments

#### Questionnaires

Differences in anxiety, stress, and depression levels between spider-fearful and non-fearful participants were assessed with the DASS (21-item version; Lovibond and Lovibond, 1995). The DASS-21 comprises three 7-item self-report scales (depression, anxiety, stress), measuring acute symptoms of depression, anxiety, and stress on a 4-point scale (0 = did not apply to me at all, 3 = applied to me very much). Sum scores for each scale as well as a total sum score were calculated for each participant. The DASS-21 has previously been associated with very good reliability estimates (Antony et al., 1998; Clara et al., 2001). Internal consistencies (Cronbach’s alpha) were in the good to excellent range: 0.88 for the depression scale, 0.82 for the anxiety scale, 0.90 for the stress scale, and 0.93 for the total scale (Henry and Crawford, 2005). Convergent and discriminant validity was good when compared with other validated measures of depression and anxiety (e.g., Hospital Anxiety and Depression Scale, Zigmond and Snaith, 1983; Personal Disturbance Scale, Bedford and Foulds, 1978; Henry and Crawford, 2005).

The FSQ [Szymanski and O’Donohue, 1995; German version by Rinck et al. (2002)] consists of 18 items depicting spider-fear-relevant statements. Agreement to each statement is rated on a 7-point scale (0 = does not apply to me at all, 6 = applies to me very much). A sum score was calculated for each participant. Internal consistency (Cronbach’s alpha) and retest-reliability of the German version of the FSQ were excellent: 0.96 and 0.95, respectively (Rinck et al., 2002).

In addition to the FSQ, the Spider Phobia Questionnaire [SPQ; Watts and Sharrock, 1984, German version by Rinck et al. (2002)] was administered to provide further information about the magnitude of spider-fear-related cognitions and avoidance behavior in spider-fearful participants. The SPQ contains 43 items describing spider-relevant situations as well as possible reactions and attitudes toward spiders. Each item is either confirmed (correct) or refused (incorrect) by the participant. A sum score was calculated. Internal consistency (Cronbach’s alpha) of the German version of the SPQ was 0.84 and retest-reliability was 0.94 (Rinck et al., 2002). Muris and Merckelbach (1996) tested both the FSQ and the SPQ and confirmed adequate reliability and validity. Both questionnaires could discriminate phobics from non-phobics, were sensitive to therapeutic change after cognitive behavior therapy, and correlated significantly with other subjective and behavioral indices of spider fear. As the FSQ and the SPQ tap somewhat different aspects of spider fear, it is recommended to administer both questionnaires in order to get a clearer picture of the nature of spider fear (Szymanski and O’Donohue, 1995; Muris and Merckelbach, 1996).

#### UCS expectancy and CS valence ratings

At the end of each phase of the differential fear conditioning paradigm, ratings of CS valence and UCS expectancy were obtained. For this purpose, each CS type was presented once again via headphones followed by a standardized, pre-recorded rating instruction that was likewise presented via headphones. Pursuant to the instruction, participants had to evaluate the valence of the particular CS (“How do you feel when you hear this tone?”) on a 5-point vertical visual analog scale ranging from −2 = very uncomfortable to +2 = very comfortable (0 = neutral). UCS expectancy (“Do you think that this tone is paired with an electrical stimulation?”) was rated from −2 = highly unlikely to +2 = most likely (0 = equiprobable).

#### Skin conductance responses

Skin conductance responses were obtained by subtracting the average SC level (SCL) during the 1000 ms preceding CS onset (baseline) from the maximum SCL recorded during the last 7 s of CS presentation. SCR data were *z*-transformed to obtain a normal distribution.

### Procedure

Upon arrival, each participant was informed about the content and goal of the experiment. In the laboratory room, participants were seated in a comfortable chair and electrodes for the measurement of SCRs as well as for the application of the electric current were attached. Together with the experimenter, each participant individually adjusted the intensity of the electric stimulation to a level they subjectively perceived as “uncomfortable but not painful” [adapted from Blechert et al. (2007)]. The experimenter explained that participants would be exposed to virtual environments via the head-mounted display while tones of different frequencies would be presented via the headphones and an electric current would be administered once in a while. Finally, the experimenter introduced and explained the vertical visual analog scale for the CS valence and UCS-expectancy rating procedure. Rating instructions were repeated and the ratings were trained with each participant several times to ensure that the rating procedure was fully understood. Thereafter, each participant was equipped with the head-mounted display and headphones, the room light was switched off, and the experimenter left the room. The experimenter controlled and monitored the experiment from the control room. CS valence and UCS-expectancy ratings were sampled online by the experimenter. After the end of the experiment, all electrodes were removed. The participants filled out the above-mentioned self-report questionnaires and were fully debriefed.

### Statistical analyses

Statistical comparisons were conducted separately for each phase (habituation, acquisition, extinction, fear retrieval) using IBM SPSS Statistics for Windows 22.0 via analyses of variance (ANOVA). For valence and UCS-expectancy ratings, the between-subjects factor group (spider-fearful vs. non-fearful) as well as the within-subjects factor CS (CS^+^ vs. CS^−^) were entered. SCRs were subjected to a group × CS × trial ANOVA, separately for the four phases. For all dependent measures, the within-subjects factor context (context A vs. context B) was added in the fear retrieval phase.

Greenhouse-Geisser correction was applied where indicated; the according (corrected) degrees of freedom are given in parentheses. The statistical significance level was set to α = 0.05. Significant main or interaction effects were followed by appropriate *post hoc* tests.

## Results

No significant differences in age and other important control variables, such as depression, stress, and anxiety levels, were evident between spider-fearful and non-fearful participants (see Table 1).

### Valence ratings

After habituation, no differences were found in valence ratings between the CS^+^ and the CS^−^ or between groups. After acquisition, a significant CS^+^/CS^−^ differentiation emerged [main effect CS; *F*_(1,41)_ = 22.91; *p* < 0.001], which was also subjected to group differences [CS × group interaction; *F*_(1,41)_ = 4.95; *p* = 0.032]: the CS^+^ was rated more negatively as compared to the CS^−^ in the spider-fearful group [*t*_(24)_ = 5.36; *p* < 0.001], but not in the non-fearful group. After extinction, no significant effects were observed. However, when both groups were tested separately, the spider-fearful group still rated the CS^+^ more negatively than the CS^−^ [*t*_(24)_ = 2.15; *p* = 0.041]; this differentiation was not seen in the non-fearful group. During the fear retrieval phase, the ANOVA with the factors CS, context, and group revealed only trends toward a main effect of the CS [*F*_(1,41)_ = 3.81; *p* = 0.058] and toward a CS × group interaction [*F*_(1,41)_ = 3.36; *p* = 0.074]. The CS^+^ was rated more negatively than the CS^−^; this was especially the case for the spider-fearful group [*t*_(24)_ = 6.77; *p* = 0.016], but not for the non-fearful group.

**Figure 2:**
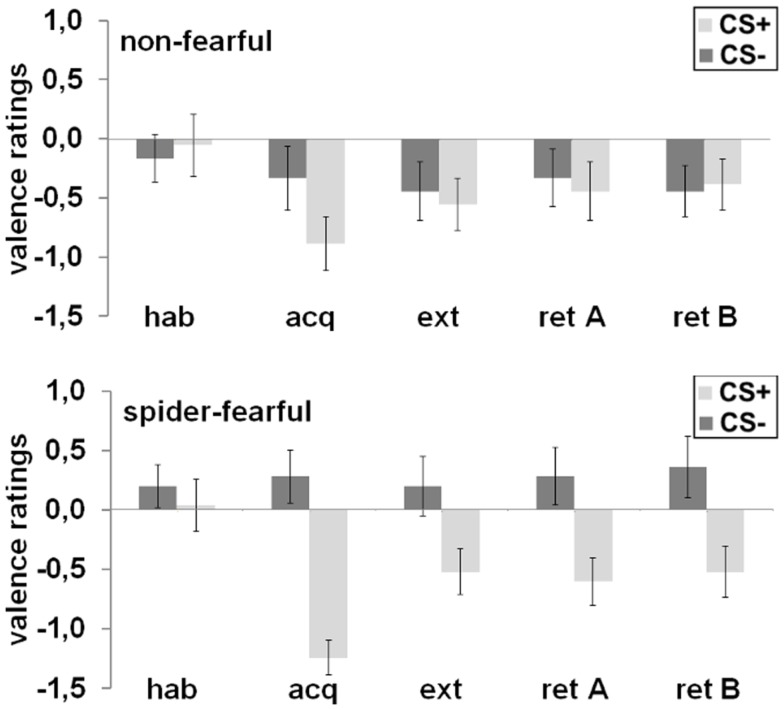
**Mean differential (CS^+^ minus CS^−^) valence ratings in the spider-fearful and non-fearful group are displayed after habituation (hab), acquisition (acq) in context A, extinction (ext) in context B, and fear retrieval in contexts A (ret A) and B (ret B)**. Error bars denote standard errors of the mean.

Taken together, spider-fearful participants reported a more negative valence toward the CS^+^ as compared to the CS^−^ after the acquisition, extinction and during the fear retrieval phase (cf. Figure 2).

### UCS-expectancy ratings

The CS^+^ and CS^−^ were not rated differently with regard to UCS expectancy after habituation. All participants rated the CS^+^ as significantly more likely to be followed by the UCS than the CS^−^ after acquisition [main effect CS; *F*_(1,41)_ = 118.49; *p* < 0.001], after extinction [main effect CS; *F*_(1,41)_ = 13.97; *p* = 0.001], and during the fear retrieval phase [main effect CS; *F*_(1,41)_ = 19.31; *p* < 0.001]. In general, the spider-fearful group stated a higher UCS expectancy during fear retrieval as compared to the non-fearful group [main effect group: *F*_(1,41)_ = 4.41; *p* = 0.042]. No other main or interaction effects were observed.

**Figure 3:**
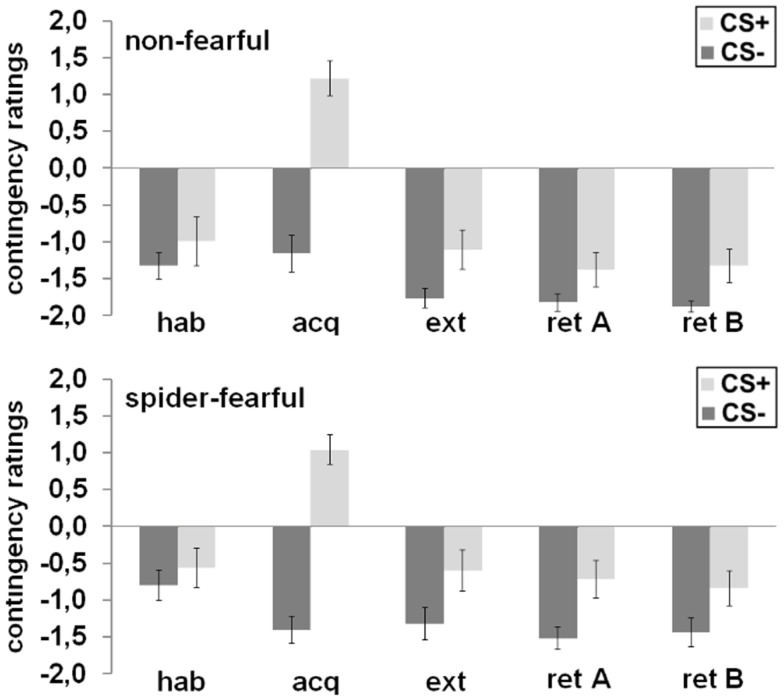
**Mean differential UCS-expectancy ratings in the spider-fearful and non-fearful group are displayed after habituation (hab), acquisition (acq) in context A, extinction (ext) in context B, and fear retrieval in contexts A (ret A) and B (ret B)**. Error bars denote standard errors of the mean.

In conclusion, spider-fearful participants only differed from non-fearful participants in their reported UCS expectancy during fear retrieval (cf. Figure 3).

### Skin conductance responses

During habituation, a main effect of trial was found [*F*_(1,41)_ = 15.87 *p* < 0.001], indicating a decrease in SCRs over the two trials. During acquisition, the main effect of trial persisted over ten trials [*F*_(6.6,268.6)_ = 2.10; *p* = 0.048]. Importantly, fear acquisition was successful as indicated by a significant differentiation between the CS^+^ and the CS^−^ [*F*_(1,41)_ = 28.31; *p* < 0.001]. Furthermore, groups differed in fear learning [CS × group interaction: *F*_(1,41)_ = 7.61; *p* = 0.009], which was driven by significantly higher SCRs toward the CS^+^ as compared to the CS^−^ in spider-fearful participants [*F*_(1,24)_ = 42.55; *p* < 0.001], but not in non-fearful persons. Additional analyses of the CS^+^ and CS^−^ trials separately showed that the spider-fearful group displayed almost significantly enhanced responding to the CS^+^ [*F*_(1,41)_ = 3.67; *p* = 0.062] and significantly attenuated responding to the CS^−^ [*F*_(1,41)_ = 6.14; *p* = 0.017] compared to the non-fearful group.

A main effect of trial occurred during extinction [*F*_(5.1,210.1)_ = 9.90; *p* < 0.001] and fear retrieval [*F*_(1.6,65.5)_ = 25.60; *p* < 0.001]. No further main or interaction effects were observed.

**Figure 4:**
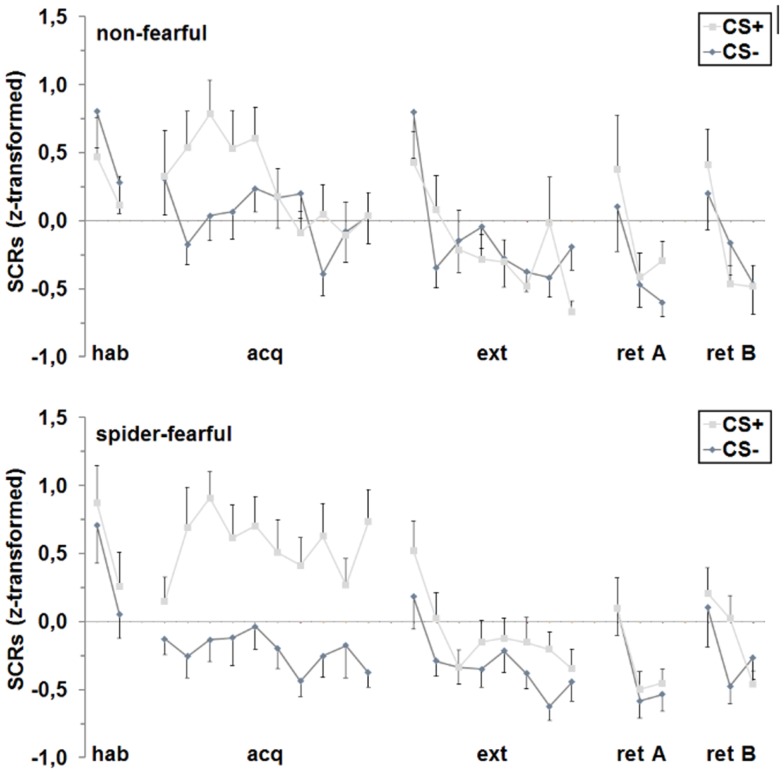
**Differential (CS^+^ minus CS^−^) SCRs for the spider-fearful and non-fearful group are shown separately for each trial of habituation (hab), acquisition (acq) in context A, extinction (ext) in context B and fear retrieval in contexts A (ret A) and B (ret B)**. Error bars denote standard errors of the mean.

Concluding, spider-fearful participants displayed higher conditioned SCRs during acquisition only, but not during the other conditioning phases (cf. Figure 4).

## Discussion

Alterations in fear acquisition and extinction have been found in patients with PD (Michael et al., 2007) and PTSD (Blechert et al., 2007). Some of these alterations seem to reflect a general deficit that is shared by both PD and PTSD, whereas other deficits might be disorder-specific. The *de novo* paradigm seems well suited to compare general conditionability across disorders. While PD and PTSD are characterized by high trait anxiety and comorbid depressive symptoms, SP is marked by fear, rather than anxiety (Grillon, 2002), of a specific object or situation, and thus seems a valuable comparison group for the investigation of shared and specific factors of fear learning in anxiety and stressor-related disorders.

In the present study, we examined changes in fear conditionability and context-dependent fear renewal in spider-fearful individuals. We found an enhanced aversive discrimination learning for *de novo* stimuli in spider-fearful individuals as evidenced on the level of electrodermal responses. This was accompanied by a more negative evaluation of the CS^+^ as compared to the CS^−^ (at the subjective valence level) in spider-fearful individuals throughout the whole conditioning procedure, i.e., the acquisition, the extinction, and the fear retrieval phase. No specific difference in extinction learning was found between spider-fearful and non-fearful participants.

Our results are in partial accordance with the propositions made by previous etiological models of anxiety disorders (Öhman and Mineka, 2001; Lissek et al., 2005). In the present study, we could demonstrate an increased capability of spider-fearful individuals to detect and respond to stimuli, which signal aversive consequences. Although we found a more negative evaluation of the CS^+^ compared to the CS^−^ in spider-fearful individuals, the SCR data suggest that superior aversive discrimination learning in spider-fearful individuals was presumably not mediated by an increased physiological responding to fear-eliciting stimuli. Hence our findings do not correspond to similar investigations in other anxiety and stressor-related disorders (e.g., Blechert et al., 2007; Michael et al., 2007; Milad et al., 2009; Norrholm et al., 2011; Jovanovic et al., 2013). For instance, we did not find evidence for increased SCR responses for CS^+^ or CS^−^ in spider-fearful individuals relative to non-fearful individuals. Thus, while spider-fearful individuals rated the CS^+^ as more negative on the subjective valence level, the physiological expression of fear (at the level of SCR) in the presence of the CS^+^ was not affected in these individuals. Conversely, the spider-fearful group rather seems to exhibit a lower threshold for the detection of cues, which signal aversive consequences and as a consequence display an enhanced fear discrimination learning.

The mechanisms underlying the enhanced fear discrimination for *de novo* fear stimuli in spider-fearful individuals remain elusive. Evidence from neurobiological studies in animals and humans suggest that the amygdala represents the most critical structure involved in the acquisition and expression of conditioned fear. Selective lesions to the amygdala impair both cued and contextual fear conditioning in animals (LeDoux, 2000). Similarly, amygdala activity increases during the acquisition relative to the extinction phase (Phelps et al., 2001; Knight et al., 2004), and there is a strong correlation between amygdala reactivity and conditioned SCRs during fear acquisition (Cheng et al., 2003; Phelps et al., 2004) in humans. The amygdala is also involved in the fast detection of potentially harming stimuli (LeDoux, 2000; Öhman and Mineka, 2001), which might represent a highly adaptive process. Spider-phobics detect and respond to phobia-relevant stimuli more rapidly (Globisch et al., 1999; Öhman et al., 2001), which might be mediated by an increased activation of the amygdalar network after confrontation with fear-related material (Dilger et al., 2003; Larson et al., 2006). This is in line with our findings on differential responding in spider-fearful individuals during the fear acquisition phase. In particular, non-fearful participants show a slight habituation of SCR during the fear acquisition phase, which is compatible with findings on habituation of amygdala activation during conditioning (LaBar et al., 1998; Phelps et al., 2001; Wright et al., 2001). In contrast, spider-fearful individuals continue to show a differential CS^+^/CS^−^ responding throughout the entire acquisition phase. This implies an exacerbated amygdalar reactivity in spider-fearful individuals associated with both the rapid detection of threatening cues as well as a lack of habituation when repeatedly confronted with these cues. Such deficient habituation of fear responses might be maladaptive in the way that pathological anxiety is maintained and further reinforced by the avoidance of cues, which signal aversive consequences (Globisch et al., 1999; Öhman et al., 2001). Interestingly, it has been reported that the hyperactivity of the amygdala that is observed in patients with SP can be normalized after successful exposure therapy (Goossens et al., 2007).

The present findings extend our knowledge on specific differences in fear acquisition and extinction between different anxiety and stressor-related disorders. Unlike to previous studies in PTSD and PD, which utilized similar methodological approaches, we did not find clear evidence for changes in fear extinction learning in spider-fearful individuals. For instance, stronger fear acquisition was found in PTSD (Orr et al., 2000), but not in patients with PD as compared to control participants (Grillon et al., 1994; Michael et al., 2007). Furthermore, PTSD but not PD patients (Michael et al., 2007) exhibited an enhanced responding to the CS^−^ during extinction (Grillon and Morgan, 1999; Peri et al., 2000; Blechert et al., 2007, 2008). This finding is interpreted as a general deficit in the ability to extract information from safety cues (Davis et al., 2000) and might represent a central feature of the PTSD psychopathology (Ehlers and Clark, 2000). Our results, by contrast, rather suggest that SP might be primarily characterized by an increased ability to discriminate between fear-related and fear-unrelated cues, which reflect the core symptomatology of SP. Namely, fear associated with specific phobias (SPs) is usually restricted to the phobic stimuli and SP exhibit an increased bias for identifying threatening material (Miltner et al., 2004). These findings are in accordance with the propositions made by “vigilance–avoidance” models of anxiety (Amir and Foa, 2001). The quick detection of aversive cues, which signal threat (which is presumably devoid of cognitive control) in SP might lead to an automatic initiation of avoidance behavior, which in turn hampers the habituation to these cues.

It should be noted, however, that the generalization of our findings warrants further replication with other measures of fear (e.g., fear-potentiated startle, neuroimaging, attention bias) to rule out the possibility that the herein observed effects are related to the specific methodology used. Nevertheless, our results imply that albeit SP, PTSD, and PD might share some common features (e.g., increased amygdalar activity, e.g., Larson et al., 2006; Etkin and Wager, 2007; Fani et al., 2012; Stevens et al., 2013), which are highly related to the symptomatology and psychopathology of these disorders, it remains at least questionable whether deficits in extinction learning represents a common biomarker of all anxiety and stressor-related disorders. Longitudinal studies could help to get more insights into the etiological role of fear learning in different anxiety and stressor-related disorders (e.g., Lommen et al., 2013).

Despite high clinical relevance, only one study so far assessed changes in conditionability in spider phobia (Schweckendiek et al., 2011). Schweckendiek et al. (2011) previously reported that, compared to healthy controls, spider-phobic patients show enhanced neuronal activations within the fear network (e.g., medial prefrontal cortex, amygdala) in response to CSs, which were paired with phobia-related pictures (UCS). Moreover, spider-phobic participants displayed higher amygdala activation in response to the phobia-related CS than to the non-phobia-related CS. The results on differences in conditionability for non-phobia-related CSs between patients and healthy controls, however, were less clear. In fact, none of the groups showed differential SCRs with respect to CSs, which were paired with non-phobia-relevant but otherwise aversive UCSs (pictures of mutilations). The authors stated that this might be attributed to the use of pictorial stimuli as UCS instead of electrical stimulation. Hence, the present findings can be considered as the first proof that – in addition to an enhanced conditionability on the neural level for phobia-relevant stimuli [see Schweckendiek et al. (2011)] – spider-fearful individuals also show an enhanced fear discrimination to phobia-irrelevant CSs. Our findings were presumably not mediated by an increased trait anxiety, concomitant increases in state depression, or changes in stress tension, since we did not find differences in these control variables between spider-fearful and non-fearful participants. Thus, consistent with previous findings, changes in cue-related anxiety responses rather than generally increased levels of anxiety (Otto et al., 2007) might be responsible for inter-individual differences in conditionability.

While spider-fearful individuals continued to rate the CS^+^ valence as negative during the fear retrieval phase, we did not observe context-induced fear renewal after extinction learning. This finding was rather unexpected and several methodical factors might account for the absence of such a finding. In the present study, we developed a modified version of an ABA fear-conditioning task and used a relatively short delay between acquisition, extinction, and fear retrieval [according to Grillon et al. (2006) and Alvarez et al. (2007)]. External context change was operationalized by VR environments. It is possible that the external context manipulation via VR technology is not suitable to reliably induce a context-dependent re-emergence of fear responses. However, given that several studies successfully demonstrated fear renewal even when using subtle changes in contextual features as an operationalization of “external context change” this assumption is quite unlikely [reviewed in Vervliet et al. (2013)]. Another explanation might be that extinction generalized across the extinction and acquisition contexts in our task because extinction was conducted shortly after acquisition [see also Myers et al. (2006)]. In this regard, it should be noted that in previous studies on human fear conditioning, the delay between the extinction phase and the renewal test was 24 h [see Maren et al. (2013)]. In the present study, where we utilized a much shorter delay, not only the association between CS^+^ and UCS might had been weakened during extinction training; but instead extinction training might also had induced a sensory habituation process to the CS^+^ stimuli as well (e.g., Lloyd et al., 2012). Thus, during the renewal test shortly after the extinction session, the CS^+^ elicited a weaker processing in the sensory system and concomitantly a weaker fear response compared to the CS^−^. This might be the reason why the renewal response is blocked after a short but not long delay between the extinction and renewal phase. The presentation of CS^+^ after 24 h in contrast might be associated with a recovery of the sensory response to the CS^+^, which in turn is more likely to induce a significant fear renewal. However, certainly more research is needed to disentangle the temporal dynamics of contextual effects on fear acquisition, extinction, and retrieval processes.

The absence of a clear clinical diagnosis for SP by means of a clinical interview in our sample of spider-fearful individuals might limit the validity of our findings. However, mean SPQ and FSQ scores in spider-fearful individuals were very high and correspond to clinical sample means (Pflugshaupt et al., 2007; Müller et al., 2011; Fisler et al., 2013; Gerdes and Alpers, 2014; Peperkorn et al., 2014; Soravia et al., 2014), suggesting that our results can be generalized to clinically significant spider phobia. Furthermore, a closer inspection of demographic data revealed that most of the spider-fearful participants indicated at least a moderate spider fear that was perceived as disturbing and accompanied by clear avoidance behavior in real life environment. Finally, the majority of spider-fearful participants were interested to participate in a future follow-up exposure therapy study with the aim to reduce their fear of spiders. However, future studies are needed to exclude the possibility that the finding of enhanced conditionability in our study is restricted to individuals who display only subclinical levels of spider fear. Although none of the participants exhibited clinically significant depressive or anxiety symptoms as evidenced from DASS scores, we cannot completely rule out that single individuals suffered from other yet undiagnosed psychiatric disease.

To our knowledge, this is the first study showing significant changes in conditionability for disorder-irrelevant stimuli in spider-fearful individuals at both the subjective and electrodermal level. Our data suggest that spider-fearful individuals show an enhanced fear discrimination while fear extinction seems to be unaffected. More research is needed, however, to understand the underlying neurobiological foundation of altered conditioning processes in spider fear. Future longitudinal studies would be valuable to provide a more causal link between altered fear learning and the development of specific fear. A better understanding of fear conditioning processes in SP and other anxiety disorders is of therapeutic significance and might help to contribute to the refinement of exposure-based treatments.

## Conflict of Interest Statement

The authors declare that the research was conducted in the absence of any commercial or financial relationships that could be construed as a potential conflict of interest.
